# Differences in the EEG Power Spectrum and Cross-Frequency Coupling Patterns between Young and Elderly Patients during Sevoflurane Anesthesia

**DOI:** 10.3390/brainsci13081149

**Published:** 2023-07-31

**Authors:** Xinxin Zhang, Ao Li, Sa Wang, Tingting Wang, Tiantian Liu, Yonghui Wang, Jingwen Fu, Guangchao Zhao, Qianzi Yang, Hailong Dong

**Affiliations:** 1 Department of Anesthesiology and Perioperative Medicine, Xijing Hospital, Fourth Military Medical University, Xi’an 710000, China; m15529332823@163.com (X.Z.); ao.li.1012@outlook.com (A.L.); wangsa2018@163.com (S.W.); 18829073775@163.com (T.W.); liusugar345@163.com (T.L.); 15902962566@163.com (Y.W.); fujingwen89@163.com (J.F.); gczhao0518@hotmail.com (G.Z.); 2 Anesthesia and Operation Center, The First Medical Center of Chinese PLA General Hospital, Beijing 100039, China; 3 Department of Anesthesiology, Ruijin Hospital, Shanghai Jiao Tong University School of Medicine, Shanghai 200025, China

**Keywords:** general anesthesia, EEG monitoring, aging brain

## Abstract

Electroencephalography (EEG) is widely used for monitoring the depth of anesthesia in surgical patients. Distinguishing age-related EEG features under general anesthesia will help to optimize anesthetic depth monitoring during surgery for elderly patients. This retrospective cohort study included 41 patients aged from 18 to 79 years undergoing noncardiac surgery under general anesthesia. We compared the power spectral signatures and phase–amplitude coupling patterns of the young and elderly groups under baseline and surgical anesthetic depth. General anesthesia by sevoflurane significantly increased the spectral power of delta, theta, alpha, and beta bands and strengthened the cross-frequency coupling both in young and elderly patients. However, the variation in EEG power spectral density and the modulation of alpha amplitudes on delta phases was relatively weaker in elderly patients. In conclusion, the EEG under general anesthesia using sevoflurane exhibited similar dynamic features between young and elderly patients, and the weakened alteration of spectral power and cross-frequency coupling patterns could be utilized to precisely quantify the depth of anesthesia in elderly patients.

## 1. Introduction

Clinical studies suggest that anesthetic exposure may induce cognitive impairment in the elderly brain. Electroencephalography (EEG) has been a popular approach for monitoring anesthetic depth and brain states noninvasively [1,2]. Several EEG-derived indices have been developed to translate raw EEG signals to specific markers of anesthetic depth, including the suppression ratio, spectral edge frequency, entropy, and bispectral index. Maintaining the bispectral index between 40 and 60 is well accepted as a suitable anesthetic depth for surgery by most anesthesia providers [2,3,4]. However, an insufficient understanding of EEG features may overestimate or underestimate the guidance of EEG for anesthetic administration in both anesthesia practice and clinical trials.

Age is a major influencing factor in the variability of EEG features across consciousness transitions [5,6]. Distinctions in EEG signatures between young and elderly individuals under general anesthesia are potentially related to the brain vulnerability of elderly patients [7]. Recent studies suggested that intraoperative alpha band power was significantly reduced in older adults with preoperative cognitive impairment and postoperative delirium (POD) [8,9]. The optimization of anesthetic exposure based on EEG monitoring was reported to ameliorate postoperative delirium (POD) and postoperative cognitive dysfunction (POCD), while some studies have suggested that this benefit could be controversial [10,11,12,13]. A possible reason is that these indices of anesthetic depth may not consider the influence of age on neural activity during anesthesia [14]. Given the higher risk of postoperative cognitive complications in older adults, a precise knowledge of EEG features in elderly individuals during anesthesia is of great importance.

A recent study showed that alpha power markedly reduced in the elderly compared to the young population during anesthesia [15], indicating the spectral differences between the young and the aged. The cross-frequency relationship is another feature of EEG oscillations apart from the spectrum, which may also play an essential role in information processing and consciousness [16]. Although previous studies reported that both propofol and sevoflurane anesthesia induced a shift in the coupling between the phase of slow oscillations (0.1–1 Hz) and the amplitude of alpha waves (8–13 Hz) when healthy adult volunteers became unconscious [17,18], the signatures of the cross-frequency coupling among the EEG bands remain unknown in either young or elderly patients.

The current study analyzed the raw prefrontal EEG signals from elderly and young patients during the wake and anesthesia maintenance periods. We compared the power spectral signatures and phase–amplitude coupling patterns of these two groups under a comparable surgical anesthetic depth. The distinctions of these EEG features may provide a new perspective for understanding the divergent responses of young and elderly brains to general anesthesia.

## 2. Materials and Methods

### 2.1. Ethical Approvement

Ethical approval for the current retrospective cohort study (KY20212092-C-1) was provided by the Medical Ethics Committee of the First Affiliated Hospital of Fourth Military Medical University, Xi’an, China on 23 June 2021. This trial was registered at Chinese Clinical Trial Registry (identifier: ChiCTR2100047879). Available Online: http://www.chictr.org.cn/ (accessed on 27 July 2023). The perioperative EEG data of patients undergoing noncardiac surgery were collected between January 2018 and January 2020.

### 2.2. Patient Selection

This study included 41 patients aged from 18 to 79 years undergoing noncardiac surgery under general anesthesia from January 2018 to January 2020. The exclusion criteria included emergency surgery, surgery involving the head and neck, anticipated difficult airway, non-Chinese speaking, or enrolment in a conflicting research study. None of the included patients had a medical history of psychiatric or neurological disorders, were under sedative or analgesic drug therapy or abuse, or had contraindication for any sedative and analgesic drugs. Patients who received propofol for induction and sevoflurane for maintenance were enrolled. Sevoflurane concentrations were captured automatically, and other drug usages were recorded manually by care providers in the electronic medical record.

### 2.3. EEG Acquisition and Preprocessing

The prefrontal EEG was continuously recorded by a ConView EEG monitor (Pearlcare Medical Technology Company Limited, Zhejiang, China) [3]. EEG monitoring consists of 4 electrodes placed over the forehead, as shown in the product manual. The earth electrode 1 was placed at Fpz, and the reference electrode 4 was placed in the temple area between the corner of the eye and hairline. The recording electrode 2 was placed over the forehead approximately at positions Fp1 or Fp2, and electrode 3 was an invalid electrode. The anesthesia index (Ai) was automatically generated by ConView and is derived from the unprocessed EEG based on sample entropy, 95% spectral edge frequency, and burst suppression ratio. Ai values were kept between 40 and 60 to maintain the anesthetic depth at a suitable level for surgeries. EEG signals (0–250 Hz) were recorded at a sampling rate of 500 Hz. The ConView monitor was already integrated into the electronic medical recording system, so that the critical events could have individual timestamps in the EEG. The preprocessing of EEG data is summarized below. Notch filters at 51 Hz and 49 Hz were applied to filter out the power line noise. EEG signals were then high-pass filtered with a cutoff frequency of 0.3 Hz using the Matlab function ‘filtfilt’ and a 4th-order Butterworth filter to avoid the potential frequency-dependent phase shifts caused by the filters. The low-pass filtering with a cutoff frequency at 50 Hz was conducted using the same 4th-order Butterworth filter and ‘filtfilt’ function. Next, motion artifacts in the EEG signals were carefully identified and removed. Specifically, the EEG signals were segmented into 10 s epochs, and the signal standard deviation in each epoch was calculated. If this value was larger than 50 μν or less than 3 μν, this epoch was considered contaminated by high-motion artifacts (e.g., eye movement artifacts) or bad electrode–tissue contact and was then excluded from further processing. In those selected epochs, a few sporadic sampling outliers could be included. These outliers were further identified and replaced by the mean if their scales were larger than four times the standard deviation of the temporal mean in the electrode. We selected 2 min EEG signals before the induction as the wake periods, and 2 min artifact-free EEG signals at the middle of the operation as the maintenance periods. The number of patients analyzed for each epoch varied due to artifact exclusion. Specifically, 16 and 18 young patients were included for analysis in the wake and maintenance periods, respectively, while 18 elderly patients were analyzed in both periods.

### 2.4. Spectral Analysis

The power spectrograms and spectrum were estimated for each subject using multitaper methods. Parameters were set using a 2 s window length with a 1.9 s overlap. The time–bandwidth product was 2, the number of Slepian tapers was 3, and the frequency resolution was 0.5 Hz. The spectrogram is a time-varying version of the power spectrum, estimated using consecutive windows of EEG data. The spectrum of frequencies throughout the entire procedure within the 0.3–50 Hz range was plotted for each patient. The spectral edge frequency was computed by averaging the frequency at which 95% of the spectral power was located across patients. The median spectrogram was related to the spectrum in which power was ranked in the middle among all the participants. We observed the power spectrum dynamics in the following specific frequency bands: delta (0.3–4 Hz), theta (4–8 Hz), alpha (8–12 Hz), beta (13–25 Hz), and gamma (26–50 Hz).

### 2.5. Phase–Amplitude Coupling Analysis (PAC)

To elicit the putative cross-frequency coupling of the EEG during the wake and anesthesia periods, we started with assessing how the amplitude of the high-frequency signal (5–30 Hz) was modulated by the phase of low-frequency oscillations (0.3–5 Hz), by constructing comodulograms [19]. In specific, EEG data were filtered to generate phase and amplitude signals, respectively. To generate phase signals, a bandpass filter (eegfilt.m in EEGLAB toolbox) was used to extract a low-frequency narrow-band signal $f_{L}(t)$. The EEG was bandpass filtered into 0.3–1.3 Hz, 1.3–2.3 Hz, 2.3–3.3 Hz, 3.3–4.3 Hz, and 4.3–5.3 Hz, respectively. To compute the amplitude signals, the EEG was bandpass filtered in 2 Hz steps between 5 and 30 Hz, each with a bandwidth of 1 Hz, and a set of 13 higher-frequency bandpass-filtered signals $f_{H}(t)$ was created. Then, the Hilbert transform was applied to extract the instantaneous phase $\psi_{L}(t)$ and instantaneous amplitude $A_{H}(t)$ from $f_{L}(t)$ and $f_{H}(t)$, respectively. For each phase/amplitude pair, the phase (range [−π, +π]) was discretized into 18 equal intervals, and each temporal $A_{H}(t)$ sample was assigned to one of 18 equally spaced phase bins based on the value of $\psi_{L}(t)$. We calculated the mean amplitude across all times in the epoch when the phase was a particular value j and denote this mean as $\langle A_{\mathrm{Hb}}\rangle (j)$, (j = 1,2,…18). The mean amplitudes were then normalized by dividing each by the sum of the mean amplitudes across all phase angle bins: $M_{b}(j)=\langle A_{\mathrm{Hb}}\rangle (j)/\sum_{j=1}^{18}\langle A_{\mathrm{Hb}}\rangle (j)$. Finally, the Kullback–Leibler distance was used to obtain the modulation index (MI) for each phase amplitude pair: $\mathrm{MI}=\sum_{j=1}^{18}M_{b}(j)\log M_{b}(j)/\log\left(18\right)+1$. If the mean amplitude is uniformly distributed over the phases, MI is measured as 0, indicating the lack of phase–amplitude coupling. MI increases when the amplitude distribution moves further away from the uniform distribution [20]. Comodulograms of MI were calculated based on a previous report [19], to determine which frequencies had a significant PAC. Then phaseampograms between the delta frequency band (0.3–2.3 Hz) and higher frequencies (5–30 Hz, 2 Hz steps) were calculated to examine whether the amplitude-modulated band resided at the peak or trough of the phase-modulated band. The MI and amplitude vector distributions were calculated on 60 s long, nonoverlapping data segments. The data were averaged to obtain a within-patient estimation. Finally, the median of the comodulograms and phaseampograms was visualized across patients.

The descriptive measures of angular data were computed by implemented functions in the CircStat toolbox [21]. The resultant vector was calculated as the mean direction in the ‘circ_mean’ function. The length of the mean resultant vector is a crucial quantity for the measurement of circular spread, and the closer the length of the mean resultant vector is to 1, the more concentrated the data sample is around the mean direction.

### 2.6. Statistical Analysis

Statistical analyses were performed using SPSS Statistics Version 28.0 (IBM Corp, Armonk, NY, USA) and customized MATLAB (MathWorks, Natick, MA, USA) codes. A Shapiro–Wilk test was applied to evaluate normality. Numerical variables with a normal distribution were presented as the mean ± SD and otherwise presented as a quartile (intervals). Categorical variables were presented as a percentage. An unpaired *t* test and Mann–Whitney U test were used for normal and non-normal variables, respectively. A Fisher exact test was used to compare categorical variables. To analyze the effect of age on EEG measures at different anesthetic stages, we conducted a two-way ANOVA analysis, and significant effects were followed-up with post hoc comparisons. A least significant difference (LSD) test was used for the multiple comparisons of intra- and intersubject factors, with significant differences at *p* < 0.05/4 (0.0125) as there are 4 comparisons for each measure, and only the adjusted *p* values (*Padj*) are reported.

## 3. Results

We analyzed the raw EEG data of 21 young patients and 20 elderly patients who underwent surgery with propofol anesthetic induction and sevoflurane maintenance. The age of the young group ranged from 20 to 38 years (30.00, 35.00) and that of the elderly group ranged from 65 to 75 years (65.25, 71.25). The demographics, clinical characteristics, and anesthetic usage of enrolled patients are summarized in Table 1. There were no significant differences between the young and elderly groups in weight, ASA, and length of surgery or anesthesia. As commonly practiced in clinical conditions, the inhaling concentrations of sevoflurane during anesthetic maintenance were controlled around one age-adjusted minimal alveolar concentration (MAC), and therefore the absolute inhaling concentrations of sevoflurane for young patients were slightly higher than for elderly patients [22].

### 3.1. Both Elderly and Young Patients Exhibited Significant Alternations in EEG Spectrograms during General Anesthesia

**Figure 1:**
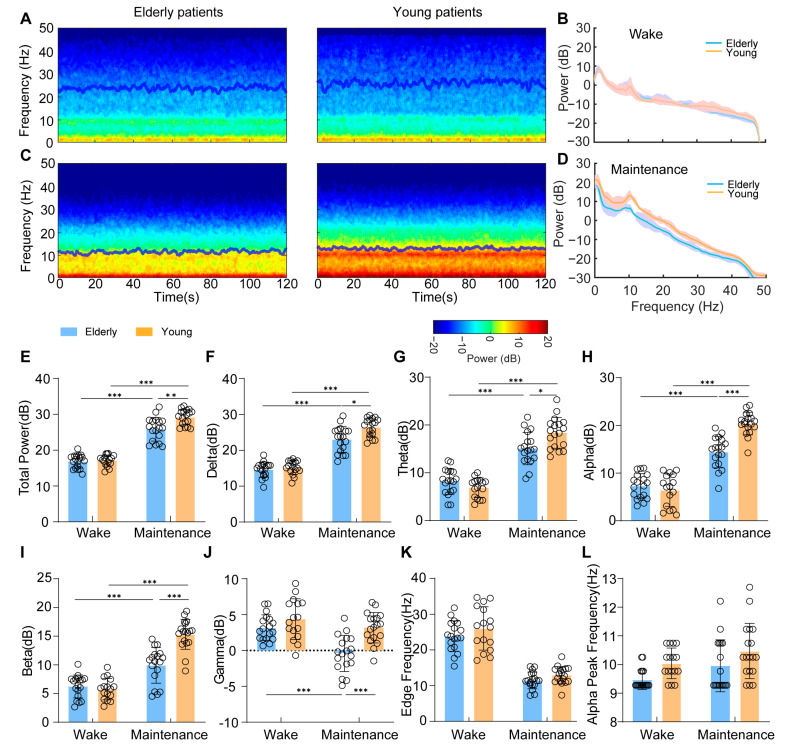
Dynamic EEG changes in both young and elderly patients during the baseline and maintenance period: (**A**) The group median spectrogram of elderly patients and young patients during the baseline; (**B**) Spectral analysis of young patients (orange line, median; shaded area, 25th–75th percentile) and elderly patients (blue line, median; shaded area, 25th–75th percentile) during the baseline; (**C**) The group median spectrogram of elderly patients and young patients during general anesthesia; (**D**) Spectral analysis of young patients (orange line, median; shaded area, 25th–75th percentile) and elderly patients (blue line, median; shaded area, 25th–75th percentile) during general anesthesia; (**E**–**L**) Change in the total power, delta, theta, alpha, beta, gamma, edge, and alpha peak frequency in elderly and young patients in the wake vs. anesthesia maintenance period. Statistical significance is indicated as * *Padj* < 0.05, ** *Padj* < 0.01, and *** *Padj* < 0.001.

In both young and elderly patients, the prefrontal EEG spectrum showed a predominant slow and alpha frequency during the baseline and anesthesia maintenance (Figure 1A–D). By analyzing with the anesthetic stage (wake or anesthetic maintenance) as the intrasubject factor and age group (young or elderly patients) as the intersubject factor, we found that EEG pattens were significantly affected by the anesthetic stage effect (Total power: F(1,66) = 355.06, *p* < 0.0001, η^2^ = 0.843; Delta: F(1,66) = 241.60, *p* < 0.0001, η^2^ = 0.785; Theta: F(1,66) = 175.03, *p* < 0.0001, η^2^ = 0.726; Alpha: F(1,66) = 227.85, *p* < 0.0001, η^2^ = 0.775; Beta: F(1,66) = 126.53, *p* < 0.0001, η^2^ = 0.657; Gamma: F(1,66) = 18.32, *p* < 0.0001, η^2^ = 0.217; Alpha peak frequency: F(1,66) = 6.76, *p* = 0.012, η^2^ = 0.093; Edge frequency: F(1,66) = 164.91, *p* < 0.0001, η^2^ = 0.720) and age effect (Total power: F(1,66) = 10.31, *p* = 0.002, η^2^ = 0.135; Delta: F(1,66) = 9.82, *p* = 0.0003, η^2^ = 0.186; Theta: F(1,66) = 2.12, *p* = 0.150, η^2^ = 0.031; Alpha: F(1,66) = 13.36, *p* = 0.001, η^2^ = 0.168; Beta: F(1,66) = 17.81, *p* < 0.0001, η^2^ = 0.212; Gamma: F(1,66) = 18.10, *p* < 0.0001, η^2^ = 0.215; Alpha peak frequency: F(1,66) = 9.48, *p* = 0.003, η^2^ = 0.126; Edge frequency: F(1,66) = 3.93, *p* = 0.052, η^2^ = 0.058). The interactions between age and stage were significant for all power bands (Total power: F(1,66) = 7.78, *p* = 0.007, $\eta^{2}$ = 0.105; Delta F(1,66) = 4.95, *p* = 0.030, $\eta^{2}$ = 0.070; Theta: F(1,66) = 10.78, *p* = 0.002, $\eta^{2}$ = 0.140; Alpha: F(1,66) = 24.49, *p* < 0.0001, $\eta^{2}$ = 0.271; Beta F(1,66) = 25.08, *p* < 0.0001, $\eta^{2}$ = 275; Gamma F(1,66) = 4.67, *p* = 0.034, $\eta^{2}$ = 0.066). Although the preanesthesia EEG spectrograms were basically comparable in two groups (Figure 1E–K; Total power: T(32) = 0.40, *Padj* = 1; Delta: T(32) = 0.83, *Padj* = 1; Theta: T(32) = 1.53, *Padj* = 1; Alpha: T(32) = 0.88, *Padj* = 1; Beta: T(32) = 0.70, *Padj* = 1; Gamma: T(32) = 1.46, *Padj* = 1), general anesthesia differentially changed the EEG features of young and elderly patients (Figure 1E–L). The total spectral power and the power of delta, theta, alpha, and beta frequencies increased after general anesthesia in both young patients (Total power: T(32) = 18.69, *Padj* < 0.0004; Delta: T(32) = 14.29, *Padj* < 0.0004; Theta: T(32) = 12.22, *Padj* < 0.0004; Alpha: T(32) = 14.11, *Padj* < 0.0004; Beta: T(32) = 12.18, *Padj* < 0.0004) and elderly patients (Total power: T(34) = 10.00, *Padj* < 0.0004; Delta: T(34) = 8.61, *Padj* < 0.0004; Theta: T(34) = 6.79, *Padj* < 0.0004; Alpha: T(34) = 7.22, *Padj* < 0.0004; Beta: T(34) = 4.22, *Padj* = 0.0008)), but the spectral power of all these bands were significant lower in the elderly patients (Total power: T(34) = 3.62, *Padj* = 0.004; Delta: T(34) = 3.26, *Padj* = 0.01; Theta: T(34) = 2.99, *Padj* = 0.02; Alpha: T(34) = 6.33, *Padj* < 0.0004; Beta: T(34) = 5.68, *Padj* < 0.0004). The alpha peak frequencies during the anesthesia maintenance period were also slightly higher than the wake period in both young and elderly patients (Figure 1L, Elderly patients: T(34) = 2.14, *Padj* = 0.1572; Young patients: T(32) = 1.58, *Padj* = 0.4940). On the contrary, the gamma band power more dramatically decreased in the elderly patients (T(34) = 4.94, *Padj* < 0.0004), and the gamma band power of young patients during the anesthesia maintenance period was also higher than elderly patients (Figure 1J, T(34) = 4.61, *Padj* < 0.0004). Due to the overall increase in lower-frequency and decrease in higher-frequency power, the spectrograms of both young and elderly patients exhibited reduced edge frequencies during general anesthesia (Figure 1K, Elderly patients: T(34) = 10.78, *Padj* < 0.0004; Young patients: T(32) = 8.45, *Padj* < 0.0004).

### 3.2. Weak Cross-Frequency Couplings Were Exhibited before General Anesthesia

**Figure 2:**
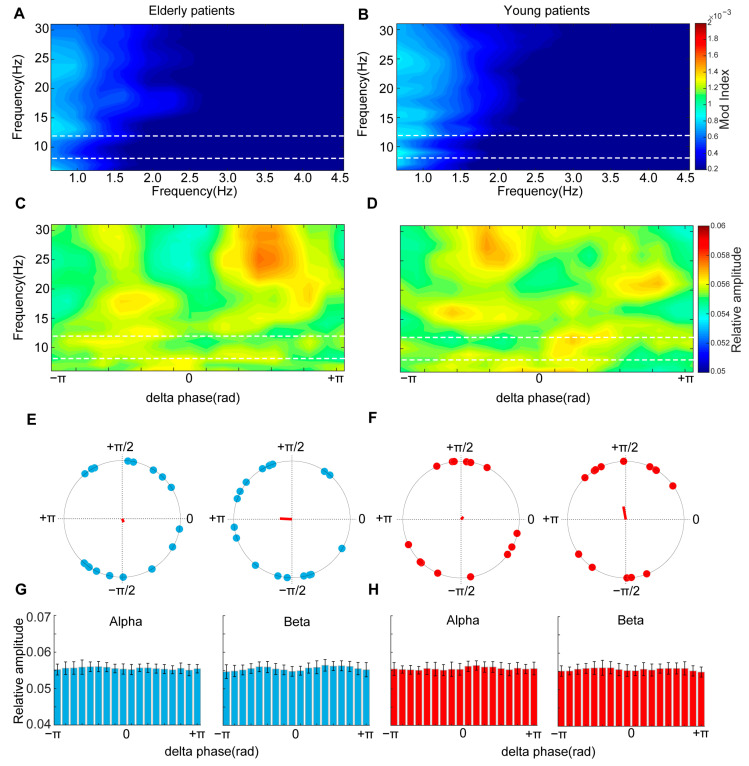
Phase–amplitude coupling patterns of young and elderly patients during the wake state: (**A**,**B**) Prefrontal comodulograms demonstrated that low-frequency oscillations were no driver of higher frequencies during wake states; (**C**,**D**) Prefrontal phaseampograms demonstrated the relative amplitude of higher frequencies was uniformly distribution; (**E**,**F**) Prefrontal circular phasor plots demonstrated that the resultant vector length was closer to 0; (**G**,**H**) Mean amplitude distribution was uniformly distributed during wake states.

Cross-frequency coupling was previously reported to be associated with brain states. Therefore, we started by comparing the comodulograms between young and elderly patients to find out the phase drivers in the pre- and intra-anesthesia states. During the wake state, the modulations were not significant in either the young or elderly group (Figure 2A,B). Considering the dominancy of the delta frequency at the unconscious states induced by general anesthesia and physiological sleep, we analyzed the amplitude distributions of higher frequencies relative to the delta phase. However, a very weak and scattered coupling was observed (Figure 2C,D). Moreover, the circular phasor plots for the delta phase and alpha or beta oscillations showed no specific phase angles of alpha/beta amplitude, and the relative amplitude of alpha/beta oscillations was uniformly distributed in the delta phase for both young (Figure 2F,H, Alpha: *p* = 0.16, Beta: *p* = 0.29, omnibus test) and elderly (Figure 2E,G, Alpha: *p* = 0.68, Beta: *p* = 0.21, omnibus test) conscious patients.

### 3.3. General Anesthesia Strengthened the Coupling of the Delta Phase with the Amplitudes of Higher-Frequency Oscillations

**Figure 3:**
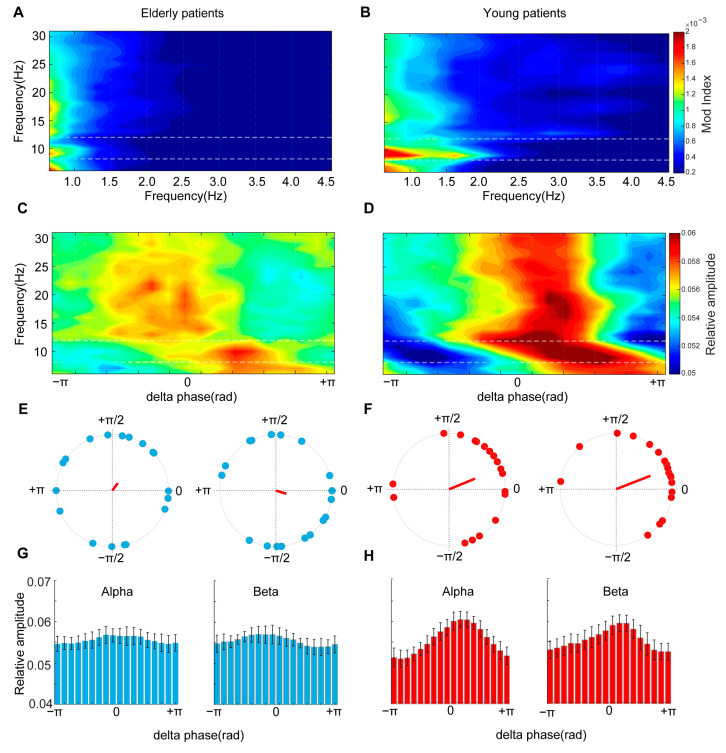
Distinct phase–amplitude coupling patterns of young and elderly patients during the maintenance period: (**A**,**B**) Prefrontal comodulograms demonstrated that delta oscillations modulated higher frequencies during sevoflurane-induced anesthetic states for both patient groups; (**C**,**D**) Prefrontal phaseampograms between delta and higher frequencies demonstrated that ‘peak-max’ patterns of phase-limited neural activity are associated anesthetic states; (**E**,**F**) Prefrontal circular phasor plots demonstrated that neural activity distributed around the 0 phase of delta oscillations, but the resultant vector length of elderly patients was smaller than in young patients; (**G**,**H**) Mean amplitude distribution was not uniformly distributed during sevoflurane-induced anesthetic states.

During the unconscious state induced by general anesthesia, the comodulogram plots showed that the delta frequency strongly modulated higher frequencies in both the young and elderly groups (Figure 3A,B). By computing phaseampograms between the delta and higher-frequency oscillations, we found that the relative amplitude of oscillations higher than 8 Hz was restricted to approximately the −π/3 to π/3 phase of delta frequencies in both the young and elderly groups (Figure 3C,D). The circular phasor plots for the delta phase and alpha frequency oscillations showed that the mean resultant vectors were approximately located in the 0 to π/3 phase in both groups (Figure 3E,F, left panels, Young patients at 22.32° and Elderly patients at 54.00°). However, the mean resultant vector of the beta frequency pointed at the 21.37° phase of delta oscillations in young patients, while at the −17.04° phase in elderly patients (Figure 3E,F, right panels). We also observed that the mean resultant vector length of both the alpha and beta frequencies for the delta oscillation phase was closer to 1 in young patients than elderly patients (Figure 3E,F, Young patients: Alpha, R = 0.50, Beta, R = 0.65; Elderly patients: Alpha, R = 0.16, Beta, R = 0.19). Consistently, the mean relative amplitudes of the alpha frequency were not uniformly distributed in the delta phase of both groups (Figure 3G,H, Young, *p* = 0.04; Elderly, *p* = 0.01, omnibus test).

### 3.4. Phase–Amplitude Couplings between the Delta Phase and Alpha/Beta Amplitude Were Impaired in Elderly Patients during General Anesthesia

**Figure 4:**
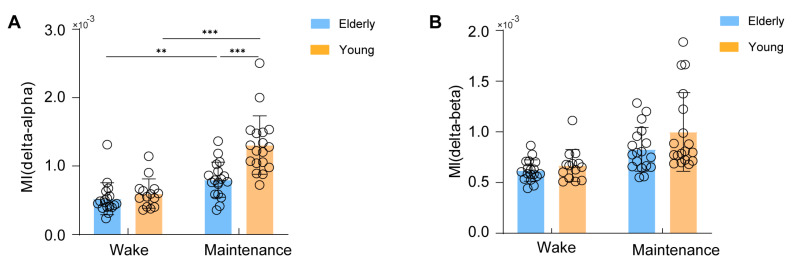
(**A**) the MI of the delta phase to alpha amplitudes in both young and elderly patients (**B**) the MI of the delta phase to beta amplitudes in both young and elderly patients. Change in MI values in elderly and young patients in the wake vs. anesthesia maintenance period. Statistical significance is indicated as ** *Padj* < 0.01, and *** *Padj* < 0.001.

To quantify the coupling of the delta phase with the amplitudes of alpha or beta oscillations, we calculated the MI values. Similar to the EEG spectrum, the MI of the delta phase to alpha amplitudes was significantly affected by both the anesthetic stage and age effect (Anesthetic stage: F(1,66) = 53.42, *p* < 0.0001, η^2^ = 0.447; age: F(1,66) = 12.37, *p* = 0.001, η^2^ = 0.158). The MI of the delta phase to alpha amplitudes during the anesthesia maintenance period was significantly higher than awake periods in both young and elderly patients (Figure 4A, Young patients: T(32) = 6.26, *Padj* < 0.0004; Elderly patients: T(34) = 3.38, *Padj* = 0.0072). Moreover, the MI of the delta phase to alpha amplitudes in young patients was significantly greater than in elderly patients, further indicating a stronger coupling of the alpha-to-delta PAC in young patients during general anesthesia maintenance (Figure 4A, T(34) = 4.33, *Padj* = 0.0004). However, the MI of the delta phase to beta amplitudes was only affected by the anesthetic stage (Figure 4B, Anesthetic stage: F(1,66) = 21.72, *p* < 0.0001, η^2^ = 0.294; age: F(1,66) = 3.68, *p* = 0.059, η^2^ = 0.053). The MI during the anesthesia maintenance period was significantly higher than awake periods in both young and elderly patients (Figure 4B, Young patients: T(32) = 3.21. *Padj* = 0.012; Elderly: T(34) = 3.69, *Padj* = 0.0032).

## 4. Discussion

EEG-guided anesthesia management provides a promising strategy to avoid unnecessary over-anesthesia or under-anesthesia application in the clinic. However, most commercial EEG indices indicating anesthetic depth do not take aging into consideration. Some studies have reported a significant difference in EEG spectral features among patients of different ages. However, the phase relationship across frequencies, another feature of composite oscillations, has been rarely investigated in distinguishing the EEG patterns during anesthesia between young and elderly patients. The current study compared the power spectral signatures and cross-frequency coupling patterns of the raw prefrontal EEG between young and elderly patients before and during general anesthesia. During the preanesthesia baseline states, young and elderly patients displayed similar spectrograms, and both groups presented weak cross-frequency couplings. Applied with a comparable clinical concentration of sevoflurane (adjusted MAC) to maintain a steady anesthesia state, elderly patients exhibited a decreased total power of EEG across all frequencies, along with a weaker ‘peak-max’ pattern in the phase–amplitude coupling (PAC) modulogram.

Revealing the spectral signatures is the primary analysis for EEG data. Consistent with the previous findings that EEG power was increased during anesthesia states [18,19], we found that the power of delta, theta, alpha, and beta frequencies increased during anesthetic states in both ages. However, overall EEG power was significantly lower in the elderly than young groups when similar age-adjusted concentrations of sevoflurane were applied to achieve the satisfied anesthetic depths for clinical surgery. The significant EEG changes could be attributed to several structural factors associated with typical aging, such as a reduction in brain volume, cortical thinning, and gray matter atrophy [5,16]. The age-related reduction in EEG power might also result from a decreased synaptic density. Consistent with previous findings, we found that the EEG spectrum of all patients showed a predominant alpha frequency during the baseline and anesthesia maintenance periods [23,24]. However, some studies have suggested that the alpha power decreased as age increased in the resting state, which we did not observe in the current study. A possible reason is that the previous reports found that the alpha power changed typically in the posterior areas instead of the prefrontal areas [25]. Moreover, the younger age of the elderly patients in our study might also lead to the discrepancy. During anesthesia, we found that the alpha power in elderly patients decreased more than other frequencies, which is consistent with the previously reported decrease of spectral power within alpha frequencies in older populations [5]. Alpha power is generated within thalamocortical circuits [26]. Cortical development during typical aging follows a ‘last to develop, first to degenerate’ pattern, which means that brain regions with high postnatal area expansion also show the most significant declines in early aging [27]. Anesthesia-induced frontal alpha follows this pattern, developing relatively late at the age of about 1 year and then receding with aging [28]. In addition, the cortical generators of propofol-induced frontal alpha oscillations appear to overlap with regions that show significant age-dependent cortical thinning [19]. Moreover, a lower EEG power in alpha frequency bands is related to POD development [29,30]. Our findings of a decreased intra-anesthesia alpha power in elderly patients further indicated the sensitive and fragile aging brain in elderly patients.

Several newly developed EEG variables have recently been used to assess conscious levels during general anesthesia [18,31,32,33]. Purdon et al. showed that the peak-max pattern between the phase of slow/delta oscillation and the amplitude of alpha oscillation could be a sign of profound unconscious states induced by general anesthetics in adult participants [17,19], indicating the cross-frequency coupling patterns may also interpret the anesthetic depth. Our study found that sevoflurane anesthesia maintained a strong coupling of the delta phase on the amplitude of higher frequencies in both the young and elderly groups. In comparison, the intensity of the modulation of delta–alpha PAC was significantly higher in young patients than elderly patients. Moreover, we found that the relative amplitude of oscillations higher than 8 Hz was restricted to approximately the −π/3 to π/3 phase of delta frequencies in young patients. This centralized alpha amplitude distribution on the delta phase, together with larger mean resultant vectors, indicated a strong peak-max PAC pattern in young patients. As for elderly patients, although the mean alpha amplitude vectors were also restricted in the 0 to π/3 phase of delta frequencies, the peak-max PAC pattern was relatively weaker. The precise spatiotemporal coordination of PAC was proven to be associated with memory consolidation [16]. Therefore, the disparate modulation intensity of the delta phase on higher-frequency oscillations between the young and elderly groups might indicate a higher incidence of postoperation cognitive deficits in elderly patients, but further investigations are needed.

Previous studies implied that potentiation of the GABAA receptor by anesthetics mediated the hyperpolarization of the thalamus and modulated the anesthesia-characterized alpha rhythm to achieve the peak-max or rise-max PAC pattern during general anesthesia [19,24,32,34]. The decreased alpha power and disturbed PAC modulation in elderly patients in the current study might be attributed to the age-related degeneration of relevant cortical and thalamus areas. Moreover, in elderly patients with pre-existing deficits in neuromodulation, the inhibition of the subcortical neural modulatory inputs by general anesthesia could further depress the metabolic interactions between astrocytes and neurons [27,35], leading to weaker oscillations and a higher tendency for burst suppression.

Electroencephalography (EEG) is widely used for monitoring brain states in the clinic, and the depth of anesthesia indices generally relies on the power or relative power of slow, delta, alpha and the percentage of suppression time to indicate unconsciousness and anesthetic depth. However, alpha oscillation power did not covary with anesthetic depth [15,18]. The delta power and relative delta power showed opposite changes along with deepening anesthesia. Moreover, spectral edge frequency in the super elderly showed no significant changes to anesthesia level, indicating the difficulties of detecting the depth of anesthesia in the super elderly by the existing EEG-derived indices [36]. Phase–amplitude coupling syntax not only tracks with the anesthesia state but also is able to distinguish the fragile brain in old adults [37], providing a promising candidate for monitoring the depth of anesthesia in elderly patients.

There are several limitations in the current study. Most enrolled elderly patients were aged 60–70 years old, which might not be able to represent the entire elderly population. Since the EEG signals were easily affected by electrosurgical interference, manual inspection was applied to choose the artifact-free epoch from the induction and maintenance periods, which may partially impede our results and conclusions. The EEG patterns of elderly patients under general anesthesia should be analyzed and compared with that of young patients in future studies with larger sample sizes or complete anesthetic trails without surgical interference. Although similar age-adjusted concentrations of sevoflurane were applied to maintain patients at appropriate states for surgical operations, the adjusted MAC values of sevoflurane were not statistically comparable; the elderly patients had a slightly higher sevoflurane MAC. Moreover, due to the lack of postanesthesia assessments of patients, the association of the proposed EEG distinctions in this study with postanesthesia complications remains to be elucidated. Future clinical studies are still needed to evaluate the predictability of these distinct EEG features throughout general anesthesia for postanesthesia neurocognitive outcomes.

## 5. Conclusions

Our study shows that elderly patients displayed different EEG features than young patients, manifested as the decreased power of multiple-frequency bands and a weaker modulation of the delta phase on the alpha amplitude. This study emphasizes the need to consider cross-frequency coupling patterns when analyzing the depth of anesthesia in elderly patients using EEG and the need for a more precise and individualized anesthetic care for elderly patients.

## Figures and Tables

**Table 1:** Patient characteristics and anesthetic adjuncts. Values are indicated by the mean (±SD). Differences were considered significant if *p* <0.05.

Variable	Young Patients (n = 21)	Elderly Patients (n = 20)	*p* Value
Age (yr)	33.00 (30.00, 35.00)	67.00 (65.25, 71.25)	<0.0001
Male (%)	23.81	40.00	0.3264
Weight (kg)	61.90 (±13.80)	62.58 (±9.82)	0.8594
ASA			0.3433
I	4 (19.05%)	1 (5%)	
II	17 (80.95%)	19 (95%)	
Length of surgery (min)	128.6 (±57.08)	153.3 (±64.01)	0.2104
Length of anesthesia (min)	167.1 (±61.33)	189.1 (±68.82)	0.2988
Induction			
Propofol (mg)	120.0 (100.0, 135.0)	100.0 (81.25,100.0)	0.0031
Rocuronium (mg)	50.00 (40.00, 50.00)	50.00 (40.00, 50.00)	0.0975
Maintenance			
Sevoflurane (%)	2.22 (2.00, 2.31)	1.87 (1.81, 1.90)	<0.0001
MAC ^1^	0.99 (0.92, 1.05)	1.06 (1.02, 1.08)	0.0013
Remifentanil (μg/kg/min)	0.15 (0.15, 0.15)	0.15 (0.15, 0.20)	0.5133
Total rocuronium (mg)	50.00 (50.00, 60.00)	50.00 (40.00, 70.00)	0.9570

^1^ Age-adjusted minimal alveolar concentration.

## Data Availability

The data presented in this study are available on request from the corresponding author.
